# Vocal Ontogeny in Neotropical Singing Mice (*Scotinomys*)

**DOI:** 10.1371/journal.pone.0113628

**Published:** 2014-12-03

**Authors:** Polly Campbell, Bret Pasch, Ashley L. Warren, Steven M. Phelps

**Affiliations:** 1 Department of Zoology, Oklahoma State University, Stillwater, Oklahoma, United States of America; 2 Bioacoustics Research Program and Macaulay Library, Cornell Lab of Ornithology, Ithaca, New York, United States of America; 3 Cornell University College of Veterinary Medicine, Ithaca, New York, United States of America; 4 Department of Integrative Biology, University of Texas at Austin, Austin, Texas, United States of America; Texas Christian University, United States of America

## Abstract

Isolation calls produced by dependent young are a fundamental form of communication. For species in which vocal signals remain important to adult communication, the function and social context of vocal behavior changes dramatically with the onset of sexual maturity. The ontogenetic relationship between these distinct forms of acoustic communication is surprisingly under-studied. We conducted a detailed analysis of vocal development in sister species of Neotropical singing mice, *Scotinomys teguina* and *S. xerampelinus*. Adult singing mice are remarkable for their advertisement songs, rapidly articulated trills used in long-distance communication; the vocal behavior of pups was previously undescribed. We recorded 30 *S. teguina* and 15 *S. xerampelinus* pups daily, from birth to weaning; 23 *S. teguina* and 11 *S. xerampelinus* were recorded until sexual maturity. Like other rodent species with poikilothermic young, singing mice were highly vocal during the first weeks of life and stopped vocalizing before weaning. Production of first advertisement songs coincided with the onset of sexual maturity after a silent period of ≧2 weeks. Species differences in vocal behavior emerged early in ontogeny and notes that comprise adult song were produced from birth. However, the organization and relative abundance of distinct note types was very different between pups and adults. Notably, the structure, note repetition rate, and intra-individual repeatability of pup vocalizations did not become more adult-like with age; the highly stereotyped structure of adult song appeared *de novo* in the first songs of young adults. We conclude that, while the basic elements of adult song are available from birth, distinct selection pressures during maternal dependency, dispersal, and territorial establishment favor major shifts in the structure and prevalence of acoustic signals. This study provides insight into how an evolutionarily conserved form of acoustic signaling provides the raw material for adult vocalizations that are highly species specific.

## Introduction

Offspring-to-parent signaling is among the most fundamental forms of communication [1]. For nocturnal mammals with altricial young (e.g., rodents, bats), acoustic signals produced by neonates (isolation calls) are integral to maternal localization and retrieval [2], [3], and can promote maternal behaviors such as grooming and nursing. Thus, across distantly related mammalian orders, both the intended receiver and the function of isolation calls seem to be conserved, and are unique to the period of maternal dependence. While vocal communication remains important to the adult social behavior of many species, the receivers and functions of acoustic signals are diverse and context-dependent, ranging from alarm calling, territorial advertisement and mate attraction, to courtship songs that encode individual identity [4], [5], [6]. The functional disconnect between neonate and adult vocal behavior raises several interesting questions. For example, are the acoustic properties of isolation calls unique or are elements retained as the animal matures and the social context and function of vocal behavior changes? Conversely, what is the developmental time course of adult vocal communication? Are adult vocalizations produced *de novo* post-weaning or can the trajectory of vocal development be traced back to isolation calls?

Available data for bats and shrews indicate that some acoustic elements of offspring to parent signals are retained in adult vocal repertoires [7], [8], [9], [10], [11]. For example, the courtship calls of adult male shrews bear a striking resemblance to contact calls produced by juveniles of both sexes [11]. Intriguingly, vocal development in lab mice is superficially similar to that in bats and shrews: while spectral and temporal features of vocalizations change with age, most syllable (note) types produced by pups reappear in the social vocalizations of adults [12], [13]. It is not clear, however, how these results relate to vocal development in wild house mice, in which the acoustic structure of adult vocalizations exhibits significant differences from that of lab mice [14]. In general, most work on acoustic communication in rodents has focused on either pups (lab mice and rats [15], [16], [17], [18]; wild species [19], [20], [21], [22], [23], [24], [25]) or adults [26], [27], [28], [29], [30], leaving the relationship between neonate and adult vocalizations largely unexplored. Importantly, studies of vocal ontogeny in wild rodent species are lacking.

Here, we characterize the vocal development of two sister species of Neotropical mice in which vocal communication plays a major role in adult social behavior. Commonly referred to as singing mice, *Scotinomys teguina* and *S. xerampelinus* are small (10–15 g) diurnal, insectivorous muroid rodents. Both species are restricted to cool, montane habitats in Middle America; *S. teguina* ranges from southern México to Panamá at 1000–2930 m, whereas *S. xerampelinus* occurs only in Costa Rica and Panamá where it replaces *S. teguina* at altitudes between 2200 and 2900 m [31].

**Figure 1:**
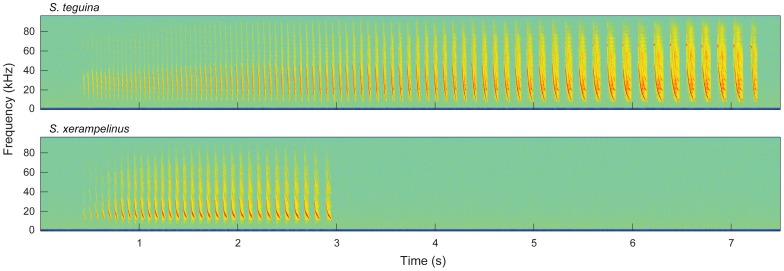
Representative spectrograms of adult advertisement songs of *S. teguina* (top) and *S. xerampelinus* (bottom).

Singing mice are remarkable for their advertisement songs, long-distance signals comprising a series of rapidly articulated frequency modulated (FM) sweeps that span audible and ultrasonic frequencies [32], [33], [34], [35]. The acoustic structure of the two species' songs is highly stereotyped: bandwidth, note duration, internote interval, and amplitude increase over the course of a song (Fig. 1). However, *S. teguina* songs are longer (typically 4–7 s vs. 1–2 s), have larger frequency bandwidths (22–43 kHz vs. 13–32 kHz), and a higher dominant frequency (22–26 kHz vs. 16–20 kHz; [29], [31], [32]). Adult *S. teguina* are typically more vocal than adult *S. xerampelinus* [32], [33]. Within species, both sexes sing but males sing significantly more than females and male songs are longer [32]. Field playback and behavioral experiments demonstrate that song is important in male–male aggression and agonistic interspecific interactions in sympatry [35]. Experiments in *S. teguina* indicate that male song is androgen-dependent and plays a role in mate attraction [33], [34]. Females show acoustic preference for male songs with faster trill rates [34], and males sing more after presentation and removal of an unfamiliar individual of the opposite sex whereas females show no such response [36].

We carried out a detailed analysis of vocal development in lab-reared *S. teguina* and *S. xerampelinus*, from birth to the emergence of advertisement songs in young adults. In all rodents in which isolation calling has been studied, neonatal vocal behavior is strongly associated with thermoregulatory capacity. Removal from the nest elicits calls from birth, with the rate of vocal production typically peaking during the first week, declining as fur grows in, and ceasing altogether around the time of first emergence from the nest. Like other muroid rodents, singing mouse pups are born deaf, blind, hairless and poikilothermic [31]. Therefore, we expected that removal from the nest would elicit vocalizations in neonates. Pup isolation calls in both species were noted by Hooper and Carleton [31], but were not quantified rigorously. The major aims of the study were to 1) trace the origin and development of the adult advertisement song of each species, 2) describe any vocal features unique to pups, and 3) determine the developmental time point at which species and sex differences in vocal characteristics and vocal behavior emerge. This is the first description of vocal ontogeny in *Scotinomys*, and is one of the few comprehensive catalogues of vocal development from birth through maturity for any rodent.

## Materials and Methods

### Experimental animals

Breeding pairs were housed in polycarbonate cages bedded with wood chips. The mice were provided with sphagnum moss and a PVC tunnel for nesting; water and food (kitten chow, seeds, dried beans and peanuts) were given *ad lib*. Pups were housed with both parents until weaning and litter sizes were not manipulated. A total of 30 *S. teguina* from 27 litters were recorded for this study; 16 of these were the offspring of wild-caught parents, captured in Cartago, Costa Rica, and 14 were the progeny of either first generation or wild-caught individuals from Boquete, Panamá. *S. xerampelinus* (*n* = 15 pups, 9 litters) were the offspring of first, second or third generation lab-reared individuals, derived from mice captured in Parque Internacional La Amistad, Panamá. All mice were housed in the same room. All animal protocols were approved by the Institutional Animal Care and Use Committee at University of Florida (No. E436).

### Recording procedure

Mice were recorded between 9:00 AM and 6:00 PM in a room acoustically isolated from the rest of the colony. The recording chamber was a polycarbonate cage bedded with clean wood chips inside a 42×42×39 cm opaque Plexiglas cube lined with anechoic foam. One side of the cube was left open to allow behavioral observations and a microphone was fitted through a small opening in the ceiling, 25 cm above the cage floor. Calls were sampled at a rate of 195 kHz, 16 bits with an ACO Pacific microphone and Tucker-Davis hardware. File sizes were set to 15 s. Fifteen seconds is >2× the average duration of adult advertisement songs [29], and reliably captured pup calling bouts in preliminary recording trials.

Subjects were brought to the recording room in their home cages. Pre-weaning pups were gently removed from their home cage and placed in the recording chamber; triggered recording began immediately as isolated pups rapidly produced vocalizations following separation. At the end of each recording period, pups were weighed with a Pesola scale and returned to their home cage. Focal neonates from each litter were initially selected at random and were identified thereafter by daily marking with indelible non-toxic magic marker until fully-furred, after which a small patch of hair was clipped. Pups were recorded for 5 minutes until both eyes opened (mean age, SD: *S. teguina* 12.1, 1.9 days; *S. xerampelinus* 19.3, 1.1 days; Table 1), and for 10 minutes from eye opening until weaning at 28–30 days. Weaned pups were housed individually and recorded for 20–30 minutes in their home cages. Most pups were recorded daily until weaning and 3–5 times a week thereafter, until they were at least 35 days old (*S. teguina*) or 39 days old (*S. xerampelinus*), and a minimum of three adult advertisement songs had been recorded. All adult songs were spontaneously produced. On average, we recorded *S. teguina* until 45 days (SD 7.1) and *S. xerampelinus* until 53 days (SD 8.9). A subset of pups (7 *S. teguina*, 4 *S. xerampelinus*) was recorded until week three only.

**Table 1:** Species, sex (F/M) and population means (SD) for litter size and developmental landmarks in singing mice.

	litter size	eyes open (d)^a^	last iso call (d)	1^st^ adult song (d)	non-calling period (d)	proportion days w/iso calls^b^
*S. teguina*	2.4 (0.90)	12.1 (1.9)	11.2 (3.4)	32.7 (8.4)	22.0 (6.8)	0.78 (0.17)
*S. teguina* B	1.6 (0.52)	10.6 (0.9)	7.5 (1.7)	26.7 (9.7)	21.5 (9.0)	0.76 (0.19)
*S. teguina* C	2.9 (0.74)	13.5 (1.4)	13.0 (2.3)	35.5 (6.2)	22.1 (6.5)	0.80 (0.16)
*S. teguina* F		11.9 (1.6)	10.1 (3.2)	34.3 (8.2)	23.5 (6.7)	0.78 (0.18)
*S. teguina* M		12.6 (2.1)	12.6 (3.2)	31.3 (8.6)	20.3 (6.9)	0.79 (0.16)
*S. xerampelinus*	2.4 (0.55)	19.3 (1.1)	9.4 (3.7)	35.0 (10.5)	25.3 (10.5)	0.56 (0.21)
*S. xerampelinus* F		19.3 (1.4)	6.3 (2.2)	34.0 (14.1)	29.0 (11.3)	0.54 (0.18)
*S. xerampelinus* M		19.3 (0.9)	11.6 (2.9)	35.4 (10.7)	23.8 (11.1)	0.59 (0.24)

a d, days;

b calculated as the number of days a pup produced isolation calls/the number of days that individual was recorded, from day 1 to the last recorded isolation call.

B Boquete;

C Cartago.

### Definitions and note categories

We use “isolation call” to refer to any vocalization produced by a pre-weaning pup that does not comprise the rapidly articulated series of FM sweeps characteristic of the adult advertisement song. We delineated the end of the isolation calling period for each individual as the last day on which an isolation call was produced. We define a note as a continuous sound, and a calling bout as a series of three or more notes occurring with an inter-note interval of less than 2 s. Notes were classified manually according to quantitative differences in duration, bandwidth, number of changes in direction of frequency (0, 1 or ≧2), and the presence/absence of nonlinear elements (e.g., deterministic chaos and bifurcations; [37]).

### Acoustic measurements

All measurements were made in Raven Pro 1.3 (Bioacoustics Research Program 2008). We analyzed a maximum of three files/individual/day and took the average of all variables. When more than three files were available we used the first three good quality files (i.e., high signal:noise) from that recording session. In each 15 s file we measured overall maximum, minimum and dominant frequency, and bandwidth. When harmonics were present, maximum and minimum frequency were always measured from the fundamental frequency (i.e., the lowest frequency in the harmonic series). We manually counted the total number of notes in each file, the number of notes in the first calling bout, and the number of notes of each type. In addition, note duration, bandwidth, and maximum, minimum and dominant frequency were measured for each of three notes in the first calling bout in the file. To avoid bias in note choice we measured bout duration, divided by three, and took measurements from notes that fell in the middle of each third (i.e., for a 6 s bout we measured the notes closest to 1.5, 3.5 and 5.5 s). Internote interval was measured from end of each focal note to the beginning of the next note in the file.

### Analysis

Principle components analysis (PCA) was used to visualize the distribution of age- and species-specific vocal parameters in acoustic space. Because the same mice were recorded at multiple ages, call data for a given individual were statistically non-independent. We controlled for non-independence in several ways. To test for age-specific differences within and between species, we binned recordings into age classes (postnatal days 1–3, 4–6, 7–9, 10–12, 13–15, 30+), and analyzed vocalizations from a single recording session for each individual within each age class. For the three-day age classes, we retained data for all individuals that were recorded in the middle of each age range (e.g., day 2 for days 1–3), and otherwise picked one representative day/individual/age class at random. Adult songs were analyzed from a single recording session/individual (mean age, SD: 40.2, 6.1 days).

Repeated measures analysis of variance (ANOVA) was used to test the effect of age on vocal development over time. In this case, only individuals that were represented in each age class were included in the analysis. To test for correlations between body mass and frequency measures from birth to maturity, we randomly assigned each individual to a focal age, such that total age range was maximized and each individual was used only once in the analysis.

We used the coefficient of variation (defined as the ratio of the standard deviation to the mean) to describe change in vocal stereotypy across age classes. Within each age class we took the average of the coefficient of variation for seven descriptors of frequency and timing (maximum, minimum and dominant frequency, number of notes/bout, bout duration, note duration for a randomly selected note in the middle third of a bout, and internote interval to the next note) from three different call files recorded from the same individual during a maximum time interval of three days (birth to 15 days) or 5 days (>30 days). In most cases, we were able to use calls captured during the same recording session. We used one-way ANOVAs to test for species and sex differences in the coefficient of variation within each age class. The α value for all statistical analyses was set to 0.05, with correction for multiple testing as appropriate.

## Results

### Vocal behavior: species, population, and sex differences

In most individuals of both species, vocal behavior was developmentally disjunct with a non-vocal period separating the last recorded isolation call and the first production of the adult advertisement song (Table 1). Of the 30 *S. teguina* pups recorded in this study, 27 produced isolation calls when removed from the nest. The three pups that never called were males derived from the Boquete population and were all from single pup litters. All 23 *S. teguina* that were recorded from birth to maturity produced adult songs, including the three males that did not call at earlier stages. Of the 15 *S. xerampelinus* pups used in the study, one female from a single pup litter did not produce isolation calls. Of the 11 individuals (5 females, 6 males) recorded to maturity, three females and one male were never observed singing as adults.

Unexpectedly, the largest differences in the duration of isolation calling and the timing of first adult song production were within *S. teguina*. On average, pups derived from the Cartago population stopped producing isolation calls almost a week later than those from Boquete (mean, SD: 13.0, 2.3 days vs. 7.5, 1.7 days; *F*_1,23_ 35.5, *P*<0.0001) and produced their first adult song over a week later than Boquete mice (35.5, 6.2 days vs. 26.7, 9.7 days; *F*_1,21_ 6.7, *P* = 0.02), whereas the duration of the non-vocal period did not differ between populations (22.1, 6.5 days vs. 21.5, 9.0 days; *F*_1,18_ 0.03, *P* = 0.9) (Table 1). We asked whether these population differences in vocal development might be a secondary consequence of different rates of growth and development, or an artifact of social or sex-specific biases in our sample.

Boquete litter sizes were smaller (*F*_1,26_ 21.6, *P*<0.0001) and Boquete pups' eyes opened earlier (*F*_1,39_ 167.0, *P*<0.0001) (Table 1). Therefore, between-population differences in vocal behavior could be explained by overall slower development in Cartago mice due to proportionally less maternal resource allocation/pup. However, there was no effect of population on body mass for any age class (all *P*≧0.2). Likewise, the end of isolation calling was three days prior to when Boquete pups opened their eyes, whereas these events were approximately coincident in Cartago pups (Table 1). We also considered that there might be an effect of litter size on motivation to vocalize; pups in this study that did not vocalize had no siblings and a similar relationship between small litter size and low isolation call production was reported in *Microtus* [22]. We could not test this hypothesis statistically since only one Cartago single-pup litter was included in the study. However, this female produced isolation calls, albeit for a period less than the population mean (8 vs. 13.0 days). Finally, the Boquete sample was strongly female-biased for all pup age classes. Therefore, if female *S. teguina* stop producing isolation calls earlier and start producing adult song later than males, population effects on vocal behavior might be an artifact of sex differences. Within the Cartago sample, however, there was no sex difference in the duration of the isolation calling period or age at first adult song production (both *P*>0.1).

These results, together with population differentiation in acoustic properties of vocalizations ([29]; described below), suggest that this bimodal pattern of vocal behavior within *S. teguina* reflects population differences in nature, which persist across several generations of lab-rearing in a common environment. To avoid confounding intraspecific variation with interspecific divergence, we carried out between-species analyses both with and without the Boquete samples. We report results for the full dataset unless exclusion of Boquete mice changed significance.

There was no difference between *S. xerampelinus* and *S. teguina* in the duration of the isolation calling period (*F*_1,37_ 2.4, *P* = 0.1), the duration of the non-vocal period (*F*_1,25_ 0.9, *P* = 0.4), or the age at which the first adult song was recorded (*F*_1,28_ 0.4, *P* = 0.6) (Table 1). With the Boquete samples excluded, however, the isolation calling period was significantly longer in *S. teguina* (*F*_1,30_ 9.4, *P* = 0.005).

Adult *S. teguina* sing longer songs and are typically more vocal than adult *S. xerampelinus*, and males of both species sing longer songs and sing more often than females [32], [35]. Therefore, we asked whether species and sex differences in vocal production are established at earlier stages. *S. teguina* pups that produced isolation calls did so on a significantly higher proportion of days than *S. xerampelinus* pups (*F*_1,36_ 12.0, *P* = 0.001) (Table 1). There was no species difference in bout length for any pup age class (all *P*≧0.3), but 1–3 day old *S. teguina* pups from Cartago tended to produce more notes/15 s than age-matched *S. xerampelinus* (*F*_1,22_ 4.0, *P* = 0.06).

Within *S. xerampelinus*, there was no sex difference in pup vocal production (proportion of days with isolation calls, bout length, note rate, all *P*≧0.1), but male pups stopped producing isolation calls significantly later than females (*F*_1,13_ 13.9, *P* = 0.003; Table 1). The small sample size for adult *S. xerampelinus* females that sang precluded tests for sex differences in the duration of the non-vocal period, or the first production of adult song. While there was no effect of sex on the proportion of days that *S. teguina* pups produced isolation calls, 4–6 and 7–9 day old *S. teguina* males produced more notes/15 s (*F*_1,18_ 4.5, *P* = 0.05 and *F*_1,18_ 5.6, *P* = 0.004, respectively) and produced longer bouts (*F*_1,18_ 4.4, *P* = 0.051 and *F*_1,18_ 8.9, *P* = 0.008, respectively).

### Vocal repertoire

**Figure 2:**
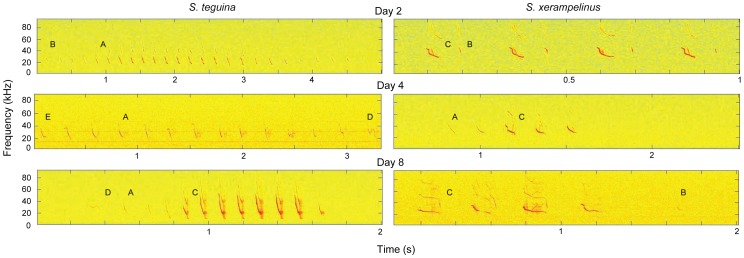
Representative spectrograms of isolation calls from age-matched *S. teguina* (left) and *S. xerampelinus* (right). Notes A-E are indicated to the right of the first note of that type in each spectrogram.

**Figure 3:**
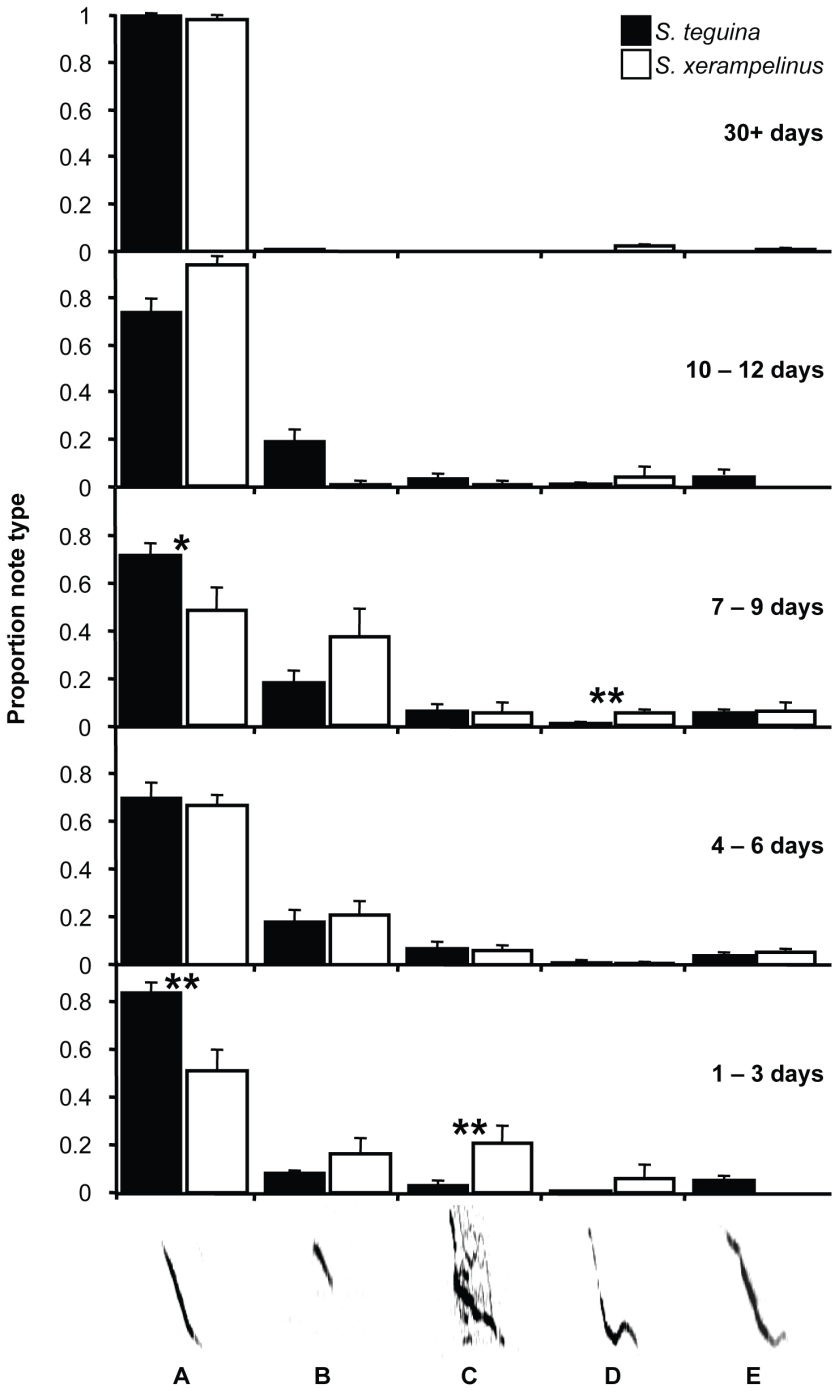
Change in the proportional abundances of common notes types during vocal development in singing mice. Representative examples of notes A–E (dominant frequency only; see Fig. 2 for harmonics) are shown on the X-axis. Black (*S. teguina*) and white (*S. xerampelinus*) bars are species means for notes in each age class. Error bars are +1 SE. Sample sizes by age class are 20 (1–3 and 30+ days), 19 (4–6 and 7–9 days) and 13 (10–12 days) for *S. teguina*, and 9 (1–3 days), 11 (4–6 days), 7 (7–9 days), 4 (10–12 days) and 8 (30+ days) for *S. xerampelinus*. Species differences in the proportional abundances of note types within age class were tested with one-way ANOVA, * *P*<0.05, **P<0.01.

We identified six distinct note types. Representative spectrograms of isolation calling bouts are shown in Figure 2; the five most common note types are shown in Figure 3.

A. Long FM downward sweep with harmonics and ≧10 kHz bandwidth. Note A is the main component of the adult advertisement song of both species.

B. Short FM downward sweep with <10 kHz bandwidth and no harmonics.

C. Notes with nonlinear components, typically an FM downward sweep combined with one or more complex elements, all with multiple harmonics and deterministic chaos.

D. Complex FM warble with harmonics and two or more directional changes in frequency ≧2 kHz.

E. Complex FM with harmonics and one directional change in frequency ≧2 kHz.

F. Atonal click.

Both species produced all note types. Developmental change and species differences in the relative proportions of A–E, the five most common note types, are plotted in Figure 3. Atonal clicks were rare in pup age classes (*S. teguina*, <0.01% notes/age class; *S. xerampelinus*, ≤5.2% notes/age class) and absent in adults. There was no interspecific difference in the mean number of note types (note diversity) produced by mice from any age class (all *P*>0.1). Note diversity in pups of both species was highest in the 4–6 day age class (mean, SD: *S. teguina* 3.3, 1.2; *S. xerampelinus* 3.7, 1.4) and lowest at the end of the isolation calling period (*S. teguina*, 13–15 days, 2.2, 1.1; *S. xerampelinus*, 10–12 days 1.8, 1.0). In both species, the long FM downward sweep (note A) characteristic of the adult advertisement song was produced from birth and was the most abundant note type in all age classes (Table S1). Note A accounted for a significantly higher proportion of *S. teguina* vs. *S. xerampelinus* vocalizations in 1–3 and 7–9 day old pups (*F*_1,28_ 14.3, *P* = 0.008 and *F*_1,25_ 5.1, *P* = 0.03, respectively), whereas *S. xerampelinus* produced a higher proportion of notes with nonlinear elements (note C) at 1–3 days (*F*_1,28_ 11.2, *P* = 0.002) and the complex FM warble (note D) at 7–9 days (*F*_1,25_ 9.1, *P* = 0.006) (Fig. 3). Note C was the only common note type that was completely absent from the adult advertisement songs of both species (Fig. 3, 30+ days). Note type proportions in males and females were statistically identical in all age classes in both species (all *P*≧0.07). Within *S. teguina*, there were minor population differences in the relative proportions of note A and the short FM downward sweep (note B). Note A was proportionally more abundant in 1–3 day old Boquete mice (*F*_1,19_ 4.4, *P* = 0.05), whereas note B was more common in Cartago mice at days 1–3 (*F*_1,19_ 9, *P* = 0.008), 4–6 (*F*_1,18_ 4.6, *P* = 0.05), and 7–9 (*F*_1,18_ 4.5, *P* = 0.05).

### Population and species effects on frequency and timing: age group comparisons

**Figure 4:**
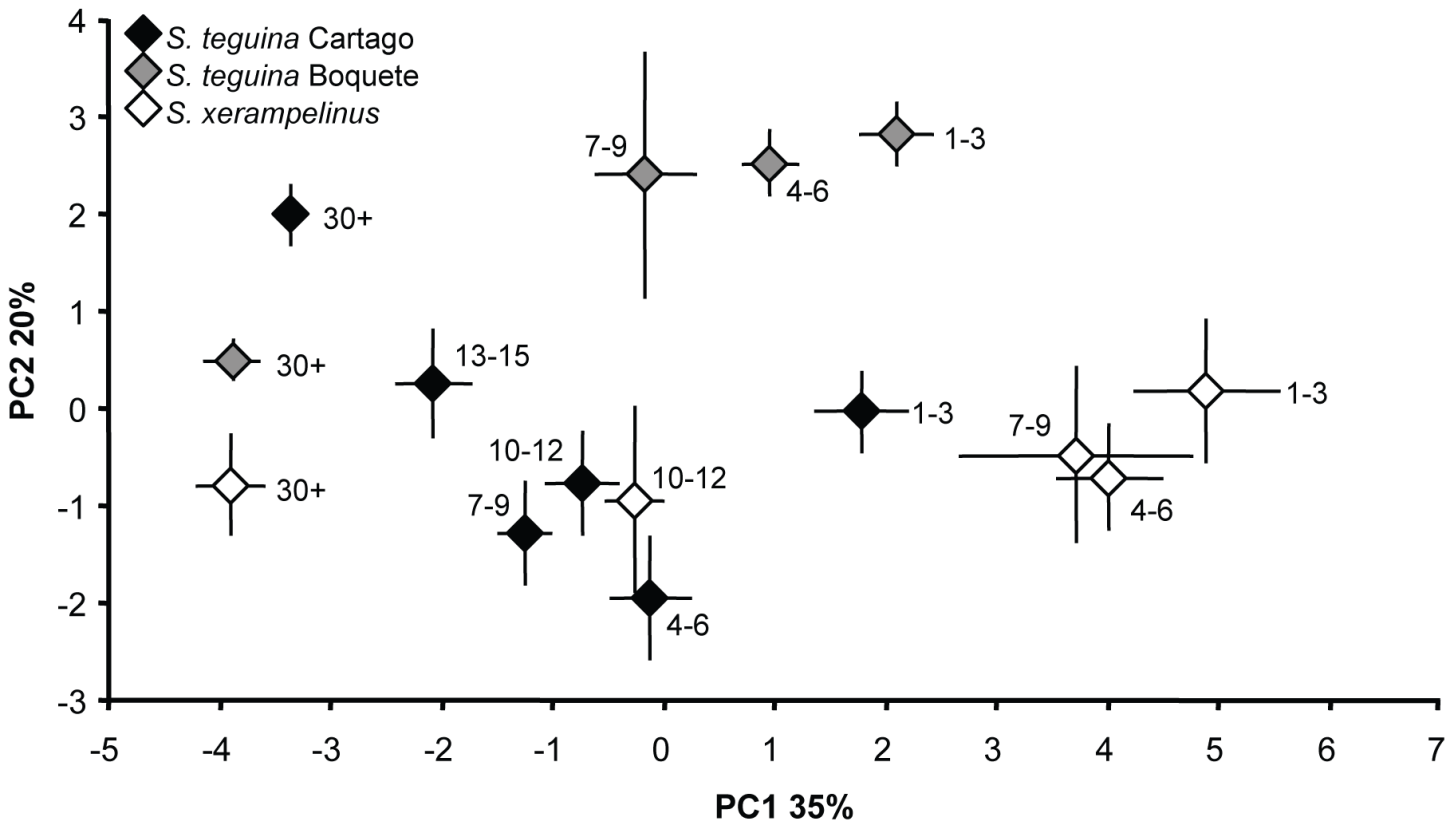
Species, population, and age differences in singing mouse vocalizations. Plot of mean scores from the first (PC1) and second (PC2) principle components axes for age classes in *S. xerampelinus* (white), and in *S. teguina* split by population (Cartago, black; Boquete, gray). Age classes are indicated next to each point. PC1 explains 35% of the total variance with higher scores corresponding to higher frequency. PC2 explains 20% of the total variance with higher scores corresponding to larger bandwidth and longer notes. Error bars are +/−1 SE.

We used PCA to visualize age, species and population differences in frequency and timing. Variables and loadings for the first two components are provided in Table S2, mean scores are plotted in Figure 4, species means for selected variables are in Table S1. All measures of frequency loaded strongly on the first axis, which accounted for 34.5% of the total variance. This axis provided strong separation between *S. teguina* and *S. xerampelinus*, and between younger pup age classes within species, with positive scores for higher frequencies. There was a clear reduction in frequency with age in both species, with the largest change over time in *S. xerampelinus*, and a striking convergence in 10–12 day old pups of both species. This axis also provided minor separation between age-matched Boquete- and Cartago-derived *S. teguina*, with higher frequencies in Boquete pups, but lower frequencies in Boquete adults. The second axis (19.6% total variance) defined differences in bandwidth and note duration with positive scores reflecting larger bandwidth and longer notes. *S. xerampelinus* age classes were largely indistinguishable along this axis, whereas there was strong separation between Boquete and Cartago *S. teguina*, with Cartago pups falling closer to age-matched *S. xerampelinus* than to Boquete conspecifics. Strikingly, while bandwidth and note duration in Cartago mice increased from 4–6 day old pups to adults, Boquete mice exhibited the opposite pattern, with a sharp decrease between 7–9 day old pups and adults. The third axis (8.2% total variance, data not shown) described among individual differences in timing and did not provide separation between species, populations or age classes.

**Figure 5:**
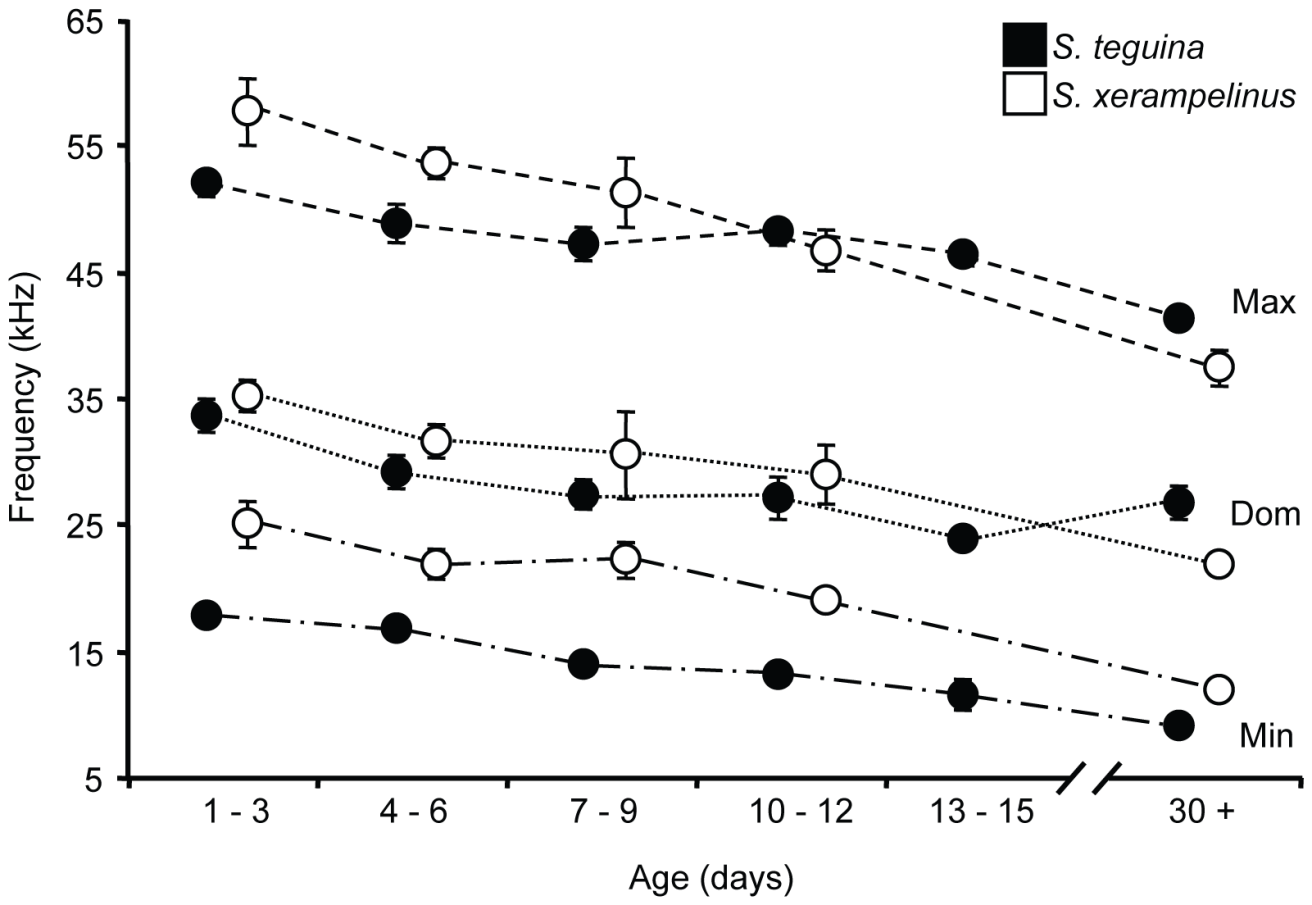
Change in call frequency during vocal development in singing mice. Black (*S. teguina*) and white (*S. xerampelinus*) circles are species means for maximum (Max, dashes), dominant (Dom, dots), and minimum (Min, dots and dashes) frequency in each age class. Error bars are +/−1 SE. Sample size for *S. teguina* in the 13–15 day age class is 9; no calls were recorded for *S. xerampelinus* in this age class. See Figure 3 caption for all other sample sizes.

Inter- and intraspecific differences in spectral measures defined by the PCA were largely recapitulated in statistical comparisons between age-matched individuals. Age-specific species means for frequency measures taken on whole calls are plotted in Figure 5, and statistics for all acoustic measures are provided in Table 2. Relative to *S. xerampelinus*, the adult songs of *S. teguina* have greater bandwidth, lower minimum frequency, and higher maximum and dominant frequency ([29], [31], [32]; Fig. 5). Species differences in the bandwidth of the adult song emerged in 10–12 day old pups, with a significantly greater frequency range in *S. teguina* (*P* = 0.0009; Table 2). In contrast, *S. teguina* pups emitted at significantly lower minimum frequencies from birth (all *P*≤0.0005; Table 2, Fig. 5). Strikingly, species differences in maximum frequency were opposite in young pups and adults. Maximum frequency was higher in young *S. xerampelinus* pups (1–3 days *P* = 0.008; 4–6 days *P* = 0.02), converged at 10–12 days, and was lower in *S. xerampelinus* adults (*P* = 0.006) (Table 2, Fig. 5). Interspecific differences in dominant frequency followed the same pattern, but decrease with age within *S. teguina* was reversed: mean dominant frequency in young adults was similar to that in 7–12 day old pups and almost 3 kHz higher than that in pups at the end of the isolation calling period (Fig. 5).

**Table 2:** Age-specific differences between *S. teguina* and *S. xerampelinus* in whole call and individual note measures of frequency and timing.

	1–3 days		4–6 days		7–9 days		10–12 days		30+ days	
	*F*_1,28_	*P*	*F*_1,29_	*P*	*F*_1,25_	*P*	*F*_1,16_	*P*	*F*_1,27_	*P*
WHOLE CALL										
Dom freq^a^									6.1	0.02^t^
Min freq^b^	28.5	**<0.0001** x	24.9	**<0.0001** x	49.8	**<0.0001** x	19.6	**0.0005** x	24.6	**<0.0001** x
Max freq^c^	8.3	0.008^x^	6.7	0.02^x^					9.2	0.006^t^
Bandwidth							16.9	**0.0009** t	22	**<0.0001** t
NOTE										
Dom freq 1			14.7	**0.0007** x	21	**0.0001** x				
Min freq 1	25.5	**<0.0001** x	20.6	**<0.0001** x	33.6	**<0.0001** x			43.2	**<0.0001** x
Max freq 1			17.1	**0.0003** x	6.2	0.02^x^				
Bandwidth 1					5.1	0.03^t^			10.8	0.003^t^
Note dur 1^d^							5.3	0.04^t^	40.2	**<0.0001** t
INI 1^e^										
INI:note dur 1^f^									19.8	0.0001^t^
Dom freq 2	11.4	**0.002** x	25.9	**<0.0001** x	19.2	**0.0002** x				
Min freq 2	28.4	**<0.0001** x	41.5	**<0.0001** x	32.3	**<0.0001** x			17.3	**0.0003** x
Max freq 2	5.1	0.03^x^	7.8	0.009^x^					11.8	**0.002** t
Bandwidth 2									17.6	**0.0003** t
Note dur 2							6.9	0.02^x^		
INI 2									6.8	0.02^t^
INI:note dur 2									16.3	**0.0004** t
Dom freq 3			25.9	**<0.0001** x	13.1	**0.0002** x				
Min freq 3	14.8	**0.0007** t	67.5	**<0.0001** t	18.6	**0.0002** t			12.1	**0.002** x
Max freq 3			10.2	0.004^x^	4.3	0.05^x^			10.6	0.003^t^
Bandwidth 3									21.1	<0.0001^t^
Note dur 3					5.5	0.03^t^	5	0.04	11.6	**0.002** x
INI 3					7.6	0.01^t^			10.8	0.003^t^
INI:note dur 3										

a dominant frequency;

b minimum frequency;

c maximum frequency;

d note duration;

e internote interval;

f note rate (ratio internote interval:note duration);

t higher value in *S. teguina*;

x higher value in *S. xerampelinus*; significance tested with ANOVA, *P*-values in bold are significant after Bonferroni correction (α = 0.002).

Interspecific differences in frequency across vocal development were generally larger than population differences within *S. teguina*. With Boquete mice excluded, *S. xerampelinus* pup dominant and maximum frequencies were marginally higher at days 4–6 (*P* = 0.03) and 7–9 (*P* = 0.02), respectively, but overall patterns were unchanged.

There were several marginal age-specific differences between *S. teguina* males and females (Table S3), but these were not consistent either within or across age groups. This general lack of sex differences in spectral measures of pup isolation calls is consistent with the sexually monomorphic spectral features of adult song in both species. However, we cannot exclude the possibility of detecting sex differences with larger sample sizes, particularly for *S. xerampelinus*.

### Relationship between body mass and frequency

In both species, there was a significant negative relationship between body mass and all whole call frequency measures (Fig. S1). In *S. teguina* (*n* = 24), the effect of body mass on dominant frequency was marginal (*F*_1,23_ 4.7, *P* = 0.04), but highly significant for minimum frequency (*F*_1,23_ 37.1, *P*<0.0001) and maximum frequency (*F*_1,23_ 16.3, *P* = 0.0006). Similarly, in *S. xerampelinus* (*n* = 13), the negative effect of body mass was weaker for dominant frequency (*F*_1,12_ 6.3, *P* = 0.03) than for minimum or maximum frequency (*F*_1,12_ 15.5, *P* = 0.002 and *F*_1,12_ 16.0, *P* = 0.002, respectively).

### Vocal change over time in *S. teguina*

We chose six variables that collectively describe frequency (dominant, minimum, maximum), note usage (proportional abundance note A), note rate (ratio internote interval:note duration measured mid-bout), and vocal behavior (bout length), and used repeated measures ANOVA to ask how these variables changed over time. The analysis was only possible for *S. teguina* (*n* = 12 mice sampled at 1–3, 4–6, 7–9, 10–12 and 30+ days, plus 2 mice with data missing for the 10–12 day age class) because there was too much missing data (i.e., recording sessions without vocalizations) for individual *S. xerampelinus*. In the full model, age had a highly significant effect on all variables except bout length (*P*≧0.008; Table 3). In pairwise comparisons between adjacent age classes, the largest changes were in the first week of life (1–3 vs. 4–6 days), and between pups near the end of the isolation calling phase and young adults.

**Table 3:** The effect of age on frequency, note usage, call length and note rate in *S. teguina*.

	Full model		1–3 vs. 4–6		4–6 vs. 7–9		7–9 vs. 10–12		10–12 vs. 30+	
Acoustic variables	*F*_1,11_	*P*	*F*_1,13_	*P*	*F*_1,13_	*P*	*F*_1,11_	*P*	*F*_1,11_	*P*
Dom freq	7.7	**0.008**	29.8	**0.0001**	0.5	0.5	1.0	0.3	1.5	0.3
Min freq	111.8	**<0.0001**	4.2	0.06	9.3	0.009	1.0	0.3	29.6	**0.0002**
Max freq	7.8	**0.007**	12.1	**0.004**	0.5	0.5	2.5	0.1	26.4	**0.0003**
Proportion note A	8.2	**0.006**	3.3	0.09	0.02	0.9	1.0	0.3	21.6	**0.0007**
Bout length	3.0	0.09	7.8	0.02	0.005	1.0	0.8	0.4	3.0	0.1
INI:note dur2	16.2	**0.0007**	5.0	0.04	0.5	0.5	0.1	0.8	12.6	**0.005**

Significance tested with repeated measures ANOVA, *P*-values in bold are significant after Bonferroni correction (α = 0.0083).

### Development of vocal stereotypy

**Figure 6:**
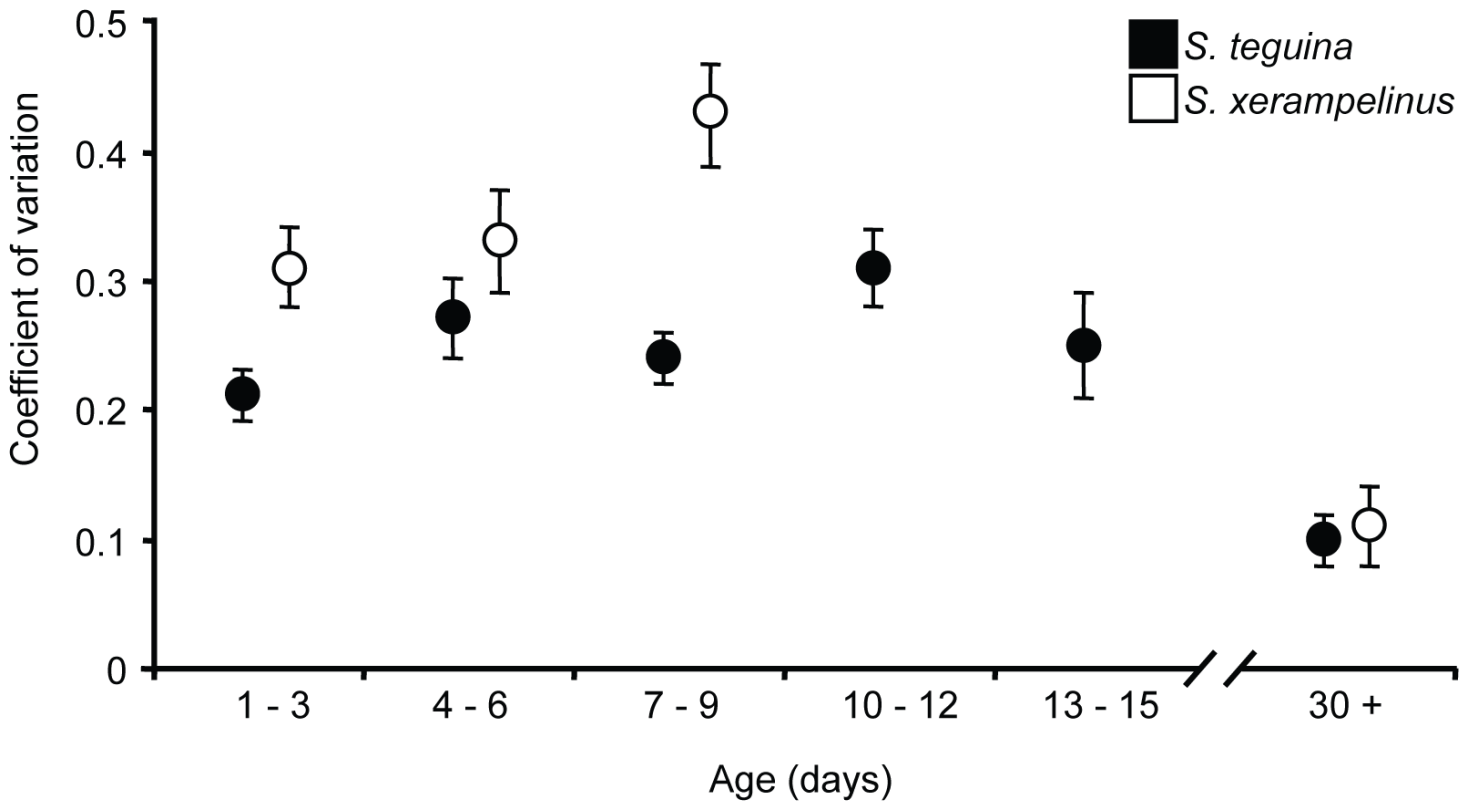
Change in stereotypy during vocal development in singing mice. Black (*S. teguina*) and white (*S. xerampelinus*) cicles are species means for the coefficient of variation in each age class. Error bars are +/−1 SE. Sample sizes by age class are 15 (1–3 days), 16 (4–6 days), 14 (7–9 days), 11 (10–12 days), 6 (13–15 days) and 18 (30+ days) for *S. teguina*, and 7 (1–3 days), 8 (4–6 days), 5 (7–9 days) and 6 (30+ days) for *S. xerampelinus*. There were insufficient data for *S. xerampelinus* in the 10–12 age class and no calls were recorded for the 13–15 age class.

The advertisement songs of adult singing mice are highly stereotyped. Therefore, we were interested in whether stereotypy increases with age as pups acquire greater motor control, or whether stereotypy is a unique feature of the adult song. For each age class, we used the coefficient of variation (CV) to summarize the within-individual repeatability of seven descriptors of frequency and timing. Species means for each age class are shown in Figure 6. Because the composition of pup vocal repertoires was heterogeneous relative to that of adults, our analysis was biased toward detecting lower stereotypy (higher CV) in pups vs. adults. Nonetheless, if increase in stereotypy were an important component of pup vocal development, we would expect to observe a reduction in intra-individual variation across pup age classes.

Contrary to this expectation, intra-individual variation increased from birth to the end of the isolation calling period in both species. However, the reduction in stereotypy with age was minor in *S. teguina* but pronounced in *S. xerampelinus*. The stereotypical properties of the adult song, evidenced by low CV, were present from the first song recorded in young adult mice (Fig. 6, 30+ days). In the subset of individuals for which we had a second series of adult songs, captured at least 10 days after the first songs we recorded, there was no evidence for an increase in stereotypy with age (*n* = 7, paired t-test, *P* = 0.3). There were no species differences in adult vocal stereotypy.

There was no effect of sex on stereotypy for any age class in either species. There was, however a species difference in age-matched pups, with higher repeatability in *S. teguina*. This difference was significant for age classes 1–3 (*F*_1,21_ 8.01, *P* = 0.01) and 7–9 (*F*_1,18_ 22.01, *P* = 0.0002).

## Discussion

We conducted a detailed analysis of vocal development in *S. teguina* and *S. xerampelinus*, species in which vocal communication is a key feature of adult social behavior. Like other rodent species with poikilothermic neonates, singing mouse pups produced isolation calls when removed from the nest, called most during the first week of life, and stopped calling well before weaning. Given these results, our main goals were to describe the relationship between pup isolation calls and the adult advertisement songs of both species, and to identify the developmental origins of species and sex differences in singing behavior, and the acoustic properties of adult song. In both species, spectral elements of adult song were clearly identifiable from birth and only 1 of 5 common note types was unique to pups. Likewise, major interspecific differences in vocal behavior and aspects of vocal timing were evident in young pups. However, with the exception of most frequency measures, acoustic properties of isolation calls did not become more adult-like as pups aged. Most notably, vocal stereotypy decreased with age in pups of both species; the highly stereotyped structure of adult advertisement songs appeared *de novo* in the first songs of young adults. Thus, while the note types that comprise the adult song of each species are produced from birth, their organization and relative abundance are very different between pups and adults. We focus our discussion of these results on the structure and function of isolation calls, the relationship between pup and adult vocalizations, and on developmental and life history correlates of vocal behavior in young adults. In closing, we consider vocal development in singing mice in relation to the much-debated capacity for vocal learning in rodents.

### The structure and function of singing mouse isolation calls

The survival of altricial neonates that are displaced from their nest depends on rapid maternal retrieval. Therefore, selection should favor isolation calls that are localizable and readily detected by adult females. In general, frequency modulated sounds are easier to localize than constant frequency sounds: directional changes in frequency enhance detectability [38], [39], and nonlinearities (e.g., noise or biphonation) are thought to prevent receiver habituation [37], [40]. In keeping with these signal design rules, most singing mouse pup vocalizations are frequency modulated and, while the unidirectional FM sweep is the most common note type in both pup isolation calls and adult advertisement song, the vocal repertoire of pups includes notes with multiple directional changes in frequency and notes with nonlinear elements, which are rare or absent in adult songs.

The major differences between *S. teguina* and *S. xerampelinus* were that the youngest age class of *S. xerampelinus* pups produced relatively more notes with nonlinearities and relatively fewer unidirectional FM sweeps, whereas *S. teguina* pups were more consistently vocal, producing isolation calls on a significantly higher proportion of days than *S. xerampelinus* pups. Given that both species produce altricial neonates, these differences suggest that eliciting rapid maternal retrieval is particularly important for newborn *S. xerampelinus*, while facilitating maternal detection and localization throughout early development is more important for *S. teguina.* The foraging and nesting behavior of lactating female singing mice is unstudied in nature; therefore we do not know why attracting their dam's attention might be particularly important for *S. xerampelinus* neonates. We speculate that greater thermoregulatory costs associated with colder ambient temperatures where *S. xerampelinus* occurs select for vocal attributes that facilitate rapid maternal retrieval, especially among poikilothermic newborns. In *S. teguina*, higher overall vocal production may be related to this species' highly developed teat-clinging behavior, which increases the probability that pups will be displaced from the nest [19], [29].

### Origins of adult vocalizations

Singing mice are unusual among muroid rodents in that long distance acoustic communication is an important modulator of adult social interactions. This motivated our focus on the ontogeny of the spectral and stereotypic properties of the adult advertisement song. However, like many species of social mammals, including other rodents, adult singing mice vocalize during close-range interactions [32]. Interestingly, complex FM warbles that resemble an elaborated version of notes D and E in young pups are the most common note type produced by adult *S. teguina* in close-range social interactions (Warren, Campbell, Pasch and Phelps unpublished data). This suggests that the distinct vocal repertoires used by adults in long-distance vs. close-range communication arise from the same source during early development. Similar patterns of vocal development, in which the heterogeneous repertoire of dependent young is modified and used in discrete contexts by adults, are reported in shrews, echolocating bats, and lab mice [10], [11], [12], [13].

Given the very different functions of acoustic signaling in pups vs. adults, it is striking that species differences in adult advertisement song follow the same pattern as that in pups: adult *S. teguina* sing longer songs and sing more often than *S. xerampelinus*. This suggests that species differences in the vocal behavior of sexually mature adults are established at an early developmental stage. While specific selection pressures such as nest predation and competition for mates and territories are unique to discrete developmental stages, species differences in ecology may select for differential investment in vocal production across ontogeny. Indeed, species differences in the structure of adult song are greater than predicted based on genetic distance data, a pattern consistent with adaptive divergence between species [29]. In *S. xerampelinus*, low resource abundance and increased thermoregulatory costs at higher, cooler altitudes may limit overall investment into acoustic communication. Similarly, a reduced ecological potential for sexual selection in resource poor environments may favor relatively lower investment in complex vocalizations by *S. xerampelinus* [41], [42].

### Proximate and ultimate correlates of vocal behavior

The onset of advertisement vocalizations following a prolonged non-vocal period coincides with important milestones in the life history of singing mice. In male *S. teguina*, testes are functional by six to eight weeks (42–56 d) of age [31]. In the present study, male *S. teguina* began producing advertisement songs by 31.3 (±8.2) days, preceding reported gamete maturation by ca. 1 week. Across mammals, release of androgens during testicular development activates a variety of appetitive behaviors used to gain access to females, including territory establishment and advertisement signaling [43]. Indeed, androgens are important activational hormones mediating vocal production and aggression in adult male singing mice; removal of the testes without exogenous androgen replacement reduces advertisement song rate and aggression, whereas gonadectomy coupled with androgen implants maintains song rate [33]. We infer that androgens link gonadal status with decisions about investment in reproductive behaviors, with songs informing conspecifics of their anticipation to mate. A similar narrative is inferred for male *S. xerampelinus*, with slight delays owing to slower developmental rates ([31]; herein).

Female *S. teguina* produced their first advertisement song (34.3±8.2 d) coincident with the onset of sexual receptivity indicated by vulval opening (33.8 d; range 28–39 d; [31]). This pattern suggests that steroid hormones associated with the estrous cycle activate female vocalizations. Androgen manipulations indicate that DHT, which cannot be aromatized to estrogen, is sufficient to activate male *S. teguina* vocalizations, suggesting that estrogens are not necessary for song production. Interestingly, the minority of males who continued to sing following castration were those castrates with the highest levels of circulating testosterone, indicating that extra-gonadal androgens may influence song output [33]. Periovulatory androgen release from the adrenals is associated with female sexual behavior in several mammalian species [43], and may activate female vocal behavior [44].

From an ultimate perspective, the non-vocal period corresponds to the span of time when pups of both species become ambulatory, are weaned, and initiate dispersal [31]. Initially, silence reflects emancipation from immobility and the ability to actively suckle by day 12 [31]. Subsequent silence in subadults likely facilitates competitor avoidance as individuals disperse in search of unoccupied habitat. Because adult vocalizations advertise signaler presence to potential rivals, remaining silent reduces the probability of escalation of costly antagonistic encounters [35]. Indeed, immigrant *S. teguina* males (who were smaller and younger) counter-sang less in response to playback of a conspecific male song compared to larger and older residents (Pasch and Phelps, unpublished data). Together, the data suggest that the emergence of adult vocalizations represents a compromise between proximate mechanisms that promote advertisement of the onset of sexual maturity, and selection that suppresses advertisement until the social environment is opportune.

### No evidence for vocal learning in singing mice

The capacity for vocal learning is rare in non-human mammals. Traditionally, rodents are classified as vocal non-learners, and recent studies using approaches such as deafening and cross-fostering indicate that the courtship songs of adult male lab mice require no auditory input for normal development and are invariant relative to the vocal environment in which animals are reared [45], [46], [47]. However, patterns of forebrain activation associated with lab mouse courtship song production, pitch convergence in songs of co-housed males from vocally distinct strains, and song degradation following deafening, suggest that the basic neural substrates and the capacity for vocal imitation (plasticity) may be present in some rodents ([48]; see also ref. 12). Arriaga and Jarvis [49] proposed that these conflicting results could be reconciled if vocal learning is treated as a continuum, placing taxa like mice with a modest capacity for vocal plasticity at one end of the spectrum, and taxa like humans and song birds with the capacity to learn and subsequently modify complex vocal output at the other.

Although our study was not designed to test for vocal learning in singing mice, three aspects of vocal development in *Scotinomys* argue against a major contribution of auditory feedback to the genesis of adult advertisement song. First, the long FM down-sweeps that comprise adult advertisement song are produced from birth. Like other altricial rodents singing mice are born deaf and call most often prior to the development of external auditory pinnae ([31]; herein). Second, vocal differences between *S. teguina* derived from different populations were maintained across several generations of captive breeding in a common environment and were evident in pre-auditory pups. This indicates that there is a substantial genetic contribution to population-specific patterns of vocal development. Third, although the rapidly articulated trills that characterize the adult advertisement songs of both species require a high degree of fine motor coordination, vocal stereotypy decreased with age in pups and the highly stereotyped structure of the advertisement song emerged *de novo* in young adults, with no apparent increase in stereotypy thereafter. Thus, there was no evidence that young singing mice practice and modify their songs relative to conspecific or internal templates.

Taken together, these observations suggest that singing mouse advertisement songs do not require extensive learning. Testing this hypothesis awaits manipulation of early acoustic environments or auditory function. Finally, given the evidence for pitch convergence in the close-range courtship songs of adult male lab mice [48], it will be of particular interest to determine whether the close-range vocal repertoires of adult singing mice are similarly sensitive to auditory feedback.

## Conclusion

The results of this study demonstrate that vocal development in sister species of singing mice follows the same basic trajectory as that described in other mammalian orders (i.e., Chiroptera and Soricomorpha; [10], [11]), and in laboratory mice [12]. While the acoustic structure, mechanistic basis, and social context of singing mouse vocalizations all undergo major shifts in the transition from isolation calls to adult advertisement songs, the basic elements of adult song are present from birth. Likewise, spectral components of isolation calls that are rare or absent in adult long distance signals are common in adult close-range interactions. This suggests that the acoustic signals of need produced by altricial mammalian neonates provide the raw material for diverse forms of adult vocal behavior and communication.

## Supporting Information

Figure S1 **The relationship between body mass and maximum (black triangles), dominant (open diamonds), and minimum (gray squares) frequency in A)** ***S. teguina*** **, and B)** ***S. xerampelinus*** **.**
(TIF)

Table S1 **Age-specific means (SD) for selected measures of frequency, timing, and vocal behavior in** ***S. teguina*** **(** ***St*** **) and** ***S. xerampelinus*** **(** ***Sx*** **).**
(DOCX)

Table S2 **Principle component (PC) axis loadings for 25 acoustic variables measured from the isolation calls and adult songs of singing mice.**
(DOCX)

Table S3 **Acoustic differences between age-matched male and female** ***S. teguina*** **.**
(DOCX)
