# Supplementary figures and images for: Vocal Ontogeny in Neotropical Singing Mice (Scotinomys)
Source: PLoS One. 2014 Dec 3;9(12):e113628. doi: 10.1371/journal.pone.0113628 (PMC4254609; doi:10.1371/journal.pone.0113628)

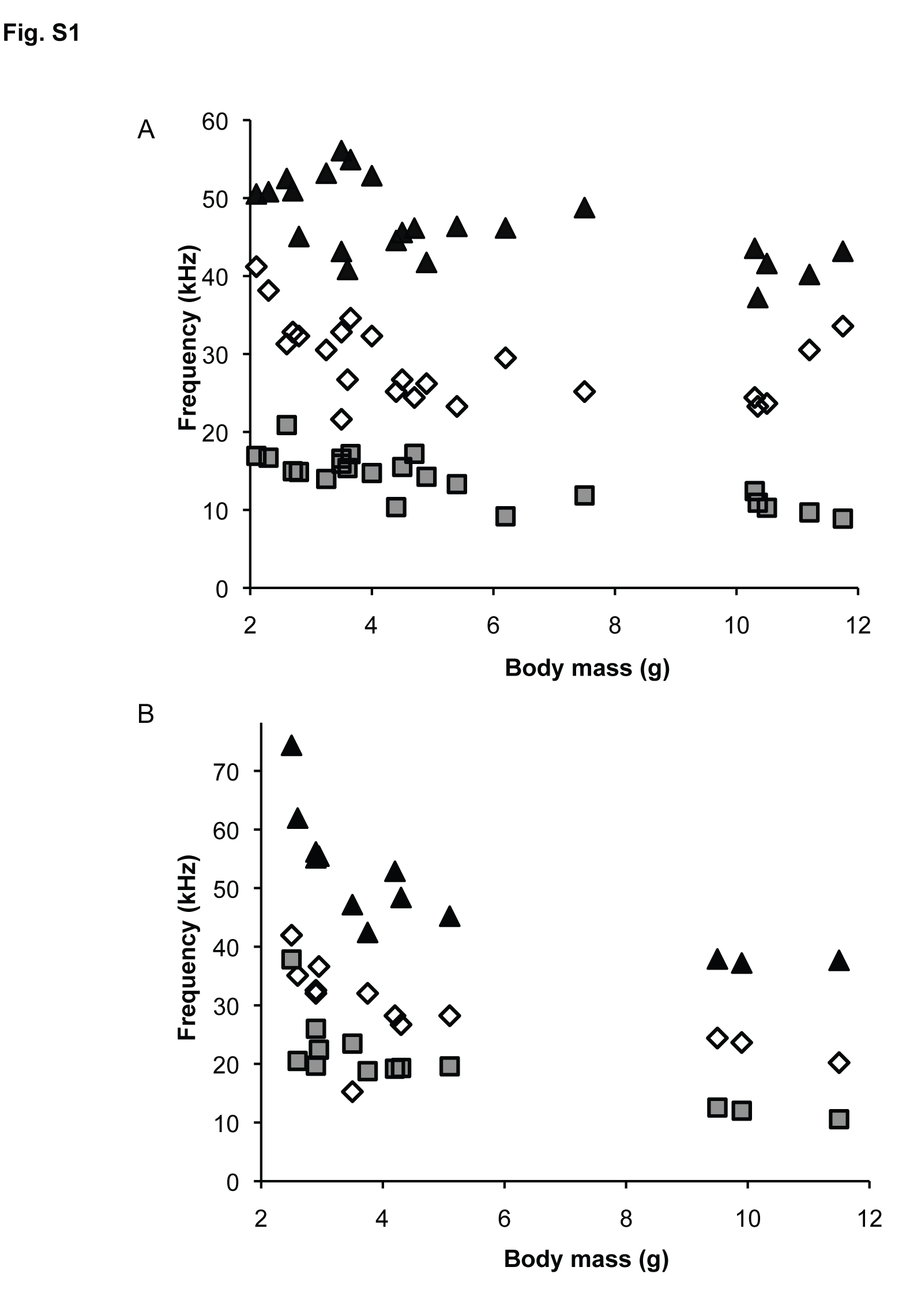

Supplement: Figure S1 — The relationship between body mass and maximum (black triangles), dominant (open diamonds), and minimum (gray squares) frequency in A) S. teguina , and B) S. xerampelinus . (TIF) [file pone.0113628.s001.tif]
