# Supplementary material for: Vocal Ontogeny in Neotropical Singing Mice (Scotinomys)
Source: PLoS One. 2014 Dec 3;9(12):e113628. doi: 10.1371/journal.pone.0113628 (PMC4254609; doi:10.1371/journal.pone.0113628)
Supplement: Table S1 — Age-specific means (SD) for selected measures of frequency, timing, and vocal behavior in S. teguina ( St ) and S. xerampelinus ( Sx ). (DOCX) [file pone.0113628.s002.docx]

Table S1. Age-specific means (SD) for selected measures of frequency, timing, and vocal behavior in *S. teguina* (*St*) and *S. xerampelinus* (*Sx*).

| Age class (days) | Species  (*n*) | Dom freq (kHz) | Min freq (kHz) | Max freq (kHz) | Bandwidth (kHz) | Proportion note A | Bout length (s) | INI:note dur 2 ^a^ |
| --- | --- | --- | --- | --- | --- | --- | --- | --- |
| 1-3 | *St* (20) | 33.6 (3.79) | 17.8 (2.10) | 52.0 (4.11) | 34.2 (3.90) | 0.84 (0.19) | 7.5 (4.71) | 5.3 (3.17) |
|  | *Sx* (9) | 35.1 (3.42) | 25.1 (5.39) | 58.4 (7.91) | 33.3 (5.50) | 0.51 (0.27) | 5.3 (4.57) | 7.8 (5.68) |
| 4-6 | *St* (19) | 29.2 (3.66) | 16.7 (5.49) | 48.7 (5.49) | 31.9 (5.68) | 0.70 (0.24) | 4.3 (3.37) | 6.5 (9.04) |
|  | *Sx* (11) | 31.6 (4.35) | 21.9 (3.13) | 53.6 (4.15) | 31.7 (5.50) | 0.67 (0.15) | 5.4 (3.13) | 6.5 (4.87) |
| 7-9 | *St* (19) | 27.3 (3.73) | 14.0 (2.14) | 47.2 (5.70) | 33.2 (6.14) | 0.71 (0.22) | 4.3 (4.160 | 5.6 (8.54) |
|  | *Sx* (7) | 30.5 (9.08) | 22.3 (3.81) | 51.3 (7.49) | 29.0 (8.14) | 0.48 (0.26) | 4.2 (3.12) | 6.1 (7.37) |
| 10-12 | *St* (13) | 27.0 (5.65) | 13.1 (2.62) | 48.2 (3.56) | 35.1 (3.75) | 0.73 (0.21) | 3.7 (4.39) | 8.3 (7.54) |
|  | *Sx* (4) | 29.0 (4.74) | 19.0 (0.70) | 45.1 (4.79) | 26.1 (4.20) | 0.93 (0.08) | 2.74 (2.11) | 8.5 (8.27) |
| 13-15 | *St* (10) | 23.9 (1.84) | 11.6 (2.72) | 46.3 (2.17) | 34.7 (3.38) | 0.90 (0.12) | 2.7 (2.25) | 3.3 (3.79) |
| 30+ | *St* (20) | 26.7 (5.24) | 9.1 (2.55) | 41.2 (2.55) | 32.2 (2.76) | 0.995 (0.012) | 5.6 (1.25) | 0.94 (0.13) |
|  | *Sx* (8) | 21.9 (2.58) | 12.0 (1.93) | 37.4 (3.97) | 25.5 (4.76) | 0.98 (0.04) | 1.5 (0.68) | 0.74 (0.08) |

*^a^* Note rate (ratio internote interval:note duration), measured in the middle third of each bout
