# Supplementary material for: Vocal Ontogeny in Neotropical Singing Mice (Scotinomys)
Source: PLoS One. 2014 Dec 3;9(12):e113628. doi: 10.1371/journal.pone.0113628 (PMC4254609; doi:10.1371/journal.pone.0113628)
Supplement: Table S2 — Principle component (PC) axis loadings for 25 acoustic variables measured from the isolation calls and adult songs of singing mice. (DOCX) [file pone.0113628.s003.docx]

Table S2. Principle component (PC) axis loadings for 25 acoustic variables measured from the isolation calls and adult songs of singing mice.

|  | Factor loading | |  |
| --- | --- | --- | --- |
| Acoustic variable | PC1 | PC2 | |
| WHOLE CALL |  |  | |
| Dom freq ^a^ | 0.659 | 0.260 | |
| Min freq ^b^ | 0.870 | -0.205 | |
| Max freq ^c^ | 0.826 | 0.360 | |
| Bandwidth | 0.201 | 0.665 | |
| NOTE |  |  | |
| Dom freq 1 | 0.830 | 0.157 | |
| Min freq 1 | 0.863 | -0.239 | |
| Max freq 1 | 0.701 | 0.424 | |
| Bandwidth 1 | -0.146 | 0.679 | |
| Note dur 1 ^d^ | 0.117 | -0.020 | |
| INI 1 ^e^ | 0.256 | -0.291 | |
| INI:note dur 1 ^f^ | 0.227 | -0.358 | |
| Dom freq 2 | 0.870 | 0.132 | |
| Min freq 2 | 0.906 | -0.287 | |
| Max freq 2 | 0.636 | 0.649 | |
| Bandwidth 2 | -0.247 | 0.852 | |
| Note dur 2 | 0.156 | 0.483 | |
| INI 2 | 0.436 | -0.161 | |
| INI:note dur 2 | 0.281 | -0.437 | |
| Dom freq 3 | 0.837 | -0.004 | |
| Min freq 3 | 0.897 | -0.275 | |
| Max freq 3 | 0.688 | 0.550 | |
| Bandwidth 3 | -0.293 | 0.768 | |
| Note dur3 | -0.087 | 0.683 | |
| INI 3 | 0.363 | -0.200 | |
| INI:note dur 3 | 0.293 | -0.345 | |
| Eigenvalue | 8.62 | 4.91 | |
| % variance explained | 34.5 | 19.6 | |

^a^ dominant frequency; ^b^ minimum frequency; ^c^ maximum frequency; ^d^ note duration; ^e^ internote interval; ^f^ note rate (ratio internote interval:note duration)
