# Supplementary material for: Vocal Ontogeny in Neotropical Singing Mice (Scotinomys)
Source: PLoS One. 2014 Dec 3;9(12):e113628. doi: 10.1371/journal.pone.0113628 (PMC4254609; doi:10.1371/journal.pone.0113628)
Supplement: Table S3 — Acoustic differences between age-matched male and female S. teguina . (DOCX) [file pone.0113628.s004.docx]

Table S3. Acoustic differences between age-matched male and female *S. teguina*

|  | 1-3 days |  | 7-9 days |  | 13-15 days |  | 30+ days |  |
| --- | --- | --- | --- | --- | --- | --- | --- | --- |
|  | *F*_1,19 (13)_ ^a^ | *P* | *F*_1,18_ | *P* | *F*_1,9_ | *P* | *F*_1,19 (13)_ | *P* |
| WHOLE CALL |  |  |  |  |  |  |  |  |
| Min freq | 5.2 | 0.04 ^M (C)^ |  |  |  |  |  |  |
| NOTE |  |  |  |  |  |  |  |  |
| High freq 1 |  |  | 5.1 | 0.04 ^F^ |  |  |  |  |
| Bandwidth 1 |  |  | 6.4 | 0.02 ^F^ |  |  |  |  |
| Note dur 1 | |  |  |  | 11.0 | 0.01 ^F^ |  |  |
| Min freq 2 |  |  |  |  |  |  | 4.9 | 0.05 ^F (C)^ |
| Bandwidth 2 | 4.9 | 0.04 ^F^ |  |  |  |  |  |  |
| Note dur 2 | |  |  |  | 8.0 | 0.02 ^F^ |  |  |
| INI:note dur 2 | 5.4 | 0.04 ^M (C)^ |  |  |  |  |  |  |

^a^ Parentheses indicate degrees of freedom for Cartago sample only; ^F^ higher value in females; ^M^ higher value in males; ^(C)^ higher in Cartago mice only; significance tested with ANOVA
